# Preclinical evaluation of high-resolution CT, ^18^F-FDG, and ^18^F-NaF PET imaging for longitudinal monitoring of atherosclerosis

**DOI:** 10.1007/s00259-025-07276-1

**Published:** 2025-04-28

**Authors:** Mikayla Tamboline, Jeffrey Collins, William Jackson, Wenduo Gu, Matthew Worssam, Paul Cheng, John David, Richard Taschereau, Arion F. Chatziioannou, Simon Jackson, Shili Xu, Oluwatayo F. Ikotun

**Affiliations:** 1 Crump Institute for Molecular Imaging, University of California, Los Angeles, Los Angeles, CA 90025 USA; 2 Department of Molecular and Medical Pharmacology, University of California, Los Angeles, Los Angeles, CA 90025 USA; 3 Department of Medicine, Division of Cardiovascular Medicine, Stanford University, Stanford, CA 94305 USA; 4 Stanford Cardiovascular Institute, Stanford University, Stanford, CA 94305 USA; 5 Cardiovascular, Amgen Inc., Thousand Oaks, CA 91320 USA; 6 Jonsson Comprehensive Cancer Center, David Geffen School of Medicine, University of California, Los Angeles, Los Angeles, CA 90025 USA; 7 Los Angeles, USA

**Keywords:** Atherosclerosis, ^18^F-NaF, ^18^F-FDG, CT, PET, Aorta, Calcification, Diagnosis

## Abstract

**Rationale:**

Detection of atherosclerosis is essential to the management and prevention of life-threatening cardiovascular events. Although non-invasive imaging modalities, such as ^18^F-sodium fluoride (^18^F-NaF), ^18^F-fluorodeoxyglucose (^18^F-FDG) PET, and CT, visualize distinct hallmarks of atherosclerosis, there has yet to be a singular multi-cohort interrogation of their strengths and limitations. Thus, we focused on identifying the optimal approach for visualizing atherosclerosis at different stages of disease progression.

**Methods:**

In this study, 6-week-old, male, ApoE deficient mice (*Apoe*^−/−^) were placed on a high cholesterol diet for 12–20 weeks to induce calcific atherosclerotic disease. Age-matched, male, wildtype (WT) C57BL/6 mice fed with regular chow served as the control group. Mice were imaged at 12, 15, 18, and 20 weeks after starting their respective diets. To follow the progression of calcified atherosclerotic lesions, at each time point, in vivo*,* ^18^F-NaF microPET/CT images were acquired 1 h and 3 h post tracer i.v. injection. In a separate cohort, in vivo ^18^F-FDG PET/CT images were acquired at 3 and 5 h post tracer i.v. injection to follow inflammation as a result of progressive atherosclerotic lesion formation. High-resolution microCT images were acquired for all mice to visualize aorta calcification. After each imaging session, a subset (*n* = 3) was euthanized from each group and histological analysis of the aorta was performed to confirm disease progression.

**Results:**

In this comparative study, within the same cohort, ^18^F-NaF PET detected atherosclerotic calcification earlier than microCT. At both 1 and 3 h post-injection (p.i.), calcified lesions were clearly detected by ^18^F-NaF with a six-fold higher signal in *Apoe*^-/-^ compared to WT mice. Interestingly, ^18^F-NaF signal peaked at week 18, whereas aortic CT signal progressively increased with a 13-, 16-, and 29-fold at 15, 18, and 20 weeks, respectively. ^18^F-FDG arortic accumulation at weeks 12 and 15, were significantly greater in *Apoe* ^−/−^ mice than WT control when images were acquired at 5 h but not at 3 h p.i.. In contrast to histological analysis, at ≥ 16 weeks where inflammation is significantly elevated, ^18^F-FDG was equivalent in *Apoe*^−/−^ and WT control mice and significantly reduced with disease progression.

**Conclusions:**

Our results show that ^18^F-NaF PET and ^18^F-FDG PET are sensitive imaging modalities for the early detection of atherosclerotic lesions. However, both ^18^F-NaF PET and high-resolution microCT prove to be effective methods for monitoring late-stage and progressive disease.

## Introduction

In the Western world, cardiovascular disease (CVD) is the underlying cause of approximately 50% of all deaths and is the predominant reason for cardiovascular events, including stroke, ischemia, and myocardial infarction [1]. The economic toll is significant worldwide; in the United States, the financial cost of CVD alone is ~ $240 billion annually [2]. Atherosclerosis is the most common contributor to cardiovascular events; with an aging population coupled with the increased prevalence of diabetes and obesity, atherosclerotic disease is predicted to exacerbate the global burden of CVD [3–6].

Atherogenesis is a complex, multifaceted, inflammatory process mediated by dynamic molecular interactions between the matrix of the artery, tissue-resident and circulatory cells, and serum constituents that regulate the formation and growth of fibroinflammatory lipid plaques [7]. Atherogenesis begins with an increase in vascular permeability that leads to the infiltration, retention, and oxidation of low-density lipoproteins (LDLs) in the subendothelial space [8]. Lipid buildup in the intimal layers leads to endothelium activation and promotes immune cell infiltration, particularly monocyte-derived macrophages that phagocytose the oxidized LDLs. Inundated with cholesterol faster than they can degrade it, infiltrated and tissue-resident macrophages become lipid-laden foam cells [9]. Foam cells contribute to the initiation and progression of atherosclerosis, and their accumulation triggers multiple pathways of programmed cell death, which in turn enlarges the necrotic cores and reduces plaque stability [9]. Accumulation of lipid-containing foam cells beneath the endothelium results in fatty streaks, the first microscopic visible signs of atherosclerosis that over time, evolve into atherosclerotic plaques [8]. As the fatty streak progresses, foam cell death and necrosis occur, and the resulting breakdown products form a highly thrombogenic lipid core covered by a collagen-rich fibrous cap that expands the plaque within the arterial wall [10].

In atherosclerosis, persistent intimal inflammation thickens the vessel walls and triggers vascular calcification [11]. Calcification, characterized by calcium phosphate deposition in the intimal and medial layers, is predictive of atherosclerotic plaque burden [10]. Calcification within the fibrous caps of atherosclerotic plaques may increase local stress, resulting in an increased risk for plaque instability and myocardial infarction [12]. Multiple imaging modalities are being developed to interrogate the compositional features of atherosclerosis. The fluorinated glucose analog, ^18^F-FDG, remains the most widely used PET tracer and has been reported for measuring plaque inflammation via the enhanced glucose consumption of activated macrophages within the aortic vessel walls [13]. Ex vivo histological assessment confirms a strong correlation between macrophage density and ^18^F-FDG uptake [4, 14, 15]. While ^18^F-FDG can assess plaque inflammation, its signal intensity is likely most pronounced in the early stages of the disease, particularly during macrophage differentiation and foam cell formation [16].

Detection and quantification of calcification surface area that is indicative of increased plaque instability can be achieved with ^18^F-NaF PET [4]. ^18^F-NaF PET provides an in vivo quantitative visualization of active vascular calcification (< 50 μm). This molecular imaging assay measures changes in the chemical composition (calcium phosphate) that precede the high-density mature calcified plaques (> 100 μm) detectable by anatomical CT imaging [4, 17]. Calcium hydroxyapatite (Ca-HAP) deposition is a key feature of vascular calcification and is the primary site of ^18^F-NaF binding, a process achieved via the displacement of exposed hydroxyl ions with radioactive fluoride ions [18]. This site for ^18^F-NaF binding enables quantitative assessment of the calcification surface area. While ^18^F-NaF PET imaging can be employed to detect areas of active calcification, CT can be utilized to measure buildup of calcified mass/density associated with the disease [19].

Using genetically engineered mouse models of atherosclerosis, preclinical investigations have reported the utility of anatomical microCT, and ^18^F-NaF or ^18^F-FDG molecular PET imaging to visualize atherosclerotic disease [4]. However, there has yet to be a holistic multi-cohort comparative analysis of the advantages and limitations of each imaging assay as a consequence of progressive disease. In this study, we comprehensively compared these translational molecular imaging methods in a mouse model of atherosclerosis and identified the imaging method optimal for each stage of the disease.

## Materials and methods

### Animals

Male apolipoprotein E deficient (*Apoe*^−/−^) 6–8 weeks old mice (Jackson Laboratory, Cat#002052) were placed on a high-fat diet (HFD) (1.25% w/w cholesterol, Research Diets #D12108 C) for 20 weeks to induce atherosclerosis disease (*n* = 40). Age-matched male wildtype (WT) C57BL/6 mice were placed on a regular chow diet and served as the experimental control (*n* = 40). All experimental protocols in this study were reviewed and approved by the Institutional Animal Care and Use Committee of the University of California, Los Angeles.

### Histological analysis

Microdissections of the mouse aorta were performed for *en* *face* Sudan IV staining of lipid to determine atherosclerotic lesion area and confirm the presence and severity of atherosclerosis. The aorta arch was dissected and cut in the coronal plane to expose the intimal surface. The dorsal descending thoracic region of the aorta was spared, the lesser curvature cut, and the aorta was folded open to reveal the intimal surface of the aortic arch. The specimen was rinsed in 70% ethanol for five minutes by positioning the aorta face-down. The specimen was then transferred to the Sudan IV working solution (1 g Sudan IV powder dissolved in a 1:1 mixture of 70% ethanol and acetone) and stained for 10 min. Followed by two washes in 80% ethanol for three minutes each. The aortic arch was then rinsed in PBS and images were acquired using a stereomicroscope connected to a digital camera at 10 times magnification [20].

### Mouse aortic root sectioning and histology

Following sacrifice, mice were perfused with 4% paraformaldehyde (PFA) solution. The mouse aortic root and proximal ascending aorta, along with the base of the heart, was excised and immersed in 4% PFA at 4 °C for 24 h. After passing through a sucrose gradient, the tissue was frozen in OCT blocks and cut into 8 µm-thick sections for immunohistochemical assessment. Immunofluorescence (IF) staining was performed following standard protocols to evaluate aortic root plaque composition [21]. Macrophage infiltrates were stained with an anti-mouse CD68 rabbit polyclonal antibody (1:300 dilution; ab125212, Abcam), followed by incubation with an AF488-conjugated secondary antibody (1:50 dilution; A21206, Invitrogen). Ferangi Blue staining was used to assess calcification, following the manufacturer’s protocol (Ferangi Blue Chromogen Kit 2, Biocare Medical, FB813). Lesion size was defined as the plaque area from the intimal edge to the border of the intima-media junction of all three cusps. Processed sections were imaged using an Echo Revolve microscope at 4 × and 10 × magnifications. Image analysis was performed using ImageJ (National Institutes of Health), using length information embedded in exported files. Statistical significance of total plaque area, CD68-positive area, and Ferangi Blue-positive area was determined using a two-sided t-test. All biological replicates were processed and stained on position-matched aortic root sections to minimize intra-experimental variance.

### ^18^F-NaF microPET/CT imaging

^18^F-NaF PET images were acquired in *Apoe*^−/−^ and WT mice at 12 (*n* = 20/group), 15 (*n* = 17/group), 18 (*n* = 14/group) and 20 (*n* = 11/group) weeks after the start of HFD or regular chow diet, respectively. Mice were anesthetized with 1.5% vaporized isoflurane followed by intravenous (i.v.) tail vein injection of 4.07 ± 0.15 MBq of ^18^F-NaF. After 60 min, mice received a 6-min PET scan (energy window 350–650 keV) followed by a microCT scan (voltage 80 kVP, current 150 µA, 720 projections, 200 µm resolution) on the GNEXT PET/CT scanner (Sofie Biosciences, Dulles, VA). Mice received a second 9-min PET scan at 3 h post tracer injection followed by a high-resolution microCT scan (voltage 80 kVP, current 150 µA, 720 projections, 100 µm resolution).

### ^18^F-FDG microPET/CT imaging

^18^F-FDG PET images were acquired on a separate cohort after 12 (*n* = 20/group), 15 (*n* = 17/group), 18 (*n* = 14/group), and 20 weeks (*n* = 11/group) on high-fat (*Apoe*^−/−^) or regular (WT) diet. Mice were injected 8.33 ± 0.09 MBq of ^18^F-FDG followed by 60 min of unconscious uptake. Each mouse was scanned at 3 h post tracer injection (static PET, 6 min) followed by a microCT scan (voltage 80 kVP, current 150 µA, 720 projections, 200 µm resolution) scan. Mice received a second PET scan (9 min) 5 h post tracer injection followed by a high-resolution CT scan (voltage 80 kVP, current 150 µA, 720 projections, 100 µm resolution).

### MicroCT imaging

To visualize calcification mass, *Apoe*^−/−^ mice and WT mice received a bin-1 high-resolution microCT scan (voltage 80 kVP, current 150 µA, 720 projections, 100 µm resolution) after 12, 15, 18, and 20 weeks on their respective diets. MicroCT data was acquired at the end of PET imaging sessions, resulting in *n* = 40/group at week 12, *n* = 34/group at week 15, *n* = 28/group at week 18, and *n* = 22/group at week 20.

### MicroPET and microCT image quantification

Acquired PET images were reconstructed using a 3D-Ordered Subset Expectation Maximization (OSEM) algorithm (24 subsets and 3 iterations), with random, attenuation, and decay corrections. MicroCT images were reconstructed using a Modified Feldkamp Algorithm. Amide software was used to analyze co-registered microPET/CT images [22]. PET data were normalized to animal body weight, and expressed in the unit of mean standardized uptake value (SUV_mean_). For ^18^F-NaF, a 5×4×2 mm region of interest (ROI) was automatically segmented above the heart using a 1-mm boundary from rib bones, and a signal intensity threshold of 0.6 SUV. For microCT, a 6×6×4 mm fixed-volume ROI and a Hounsfield Unit (HU) threshold of 350 (HU water = 0) was placed in the thoracic cavity. The volumetric calcium content of the aorta (vHU) was defined as the product of mean ROI (HU) and the ROI volume (mm^3^) summed over all ROIs for each mouse [23]. For ^18^F-FDG images, ROIs for the aorta were obtained by drawing the ROI over the aorta through each slice of the scan.

### Statistical analysis

Statistical analyses were performed using GraphPad Prism (Version 10.4.1). Two-way ANOVA analysis was performed to determine differences between the two cohorts (WT control vs. *Apoe*^*−/−*^ on HFD) in the longitudinal study, combining the effect of group and time on the biomarkers. Tukey’s multiple comparison was used for between-group comparisons, and statistical significance was set at a *p*-value ≤ 0.05. One-way ANOVA was used for within-group analysis with Šídák's multiple comparisons test to determine statistical significance. Correlative analysis was conducted using the Pearson’s rank correlation coefficient and the r^2^ and *p*-values reported.

## Results

### Establishing an atherosclerosis mouse model and confirming disease progression

**Fig. 1:**
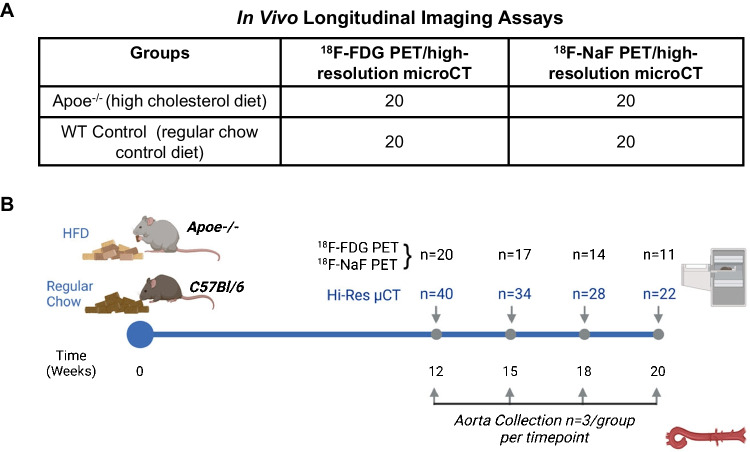
Experimental study design and timeline of longitudinal in vivo imaging assays. **A** Two groups of mice, *Apoe*^−/−^ on a high fat diet (HFD) and C57BL/6 wildtype control mice on a regular diet, were imaged with two microPET tracers: ^18^F-NaF (*n* = 20/group) and a separate cohort imaged by ^18^F-FDG (*n* = 20/group). MicroCT was performed on both cohorts combined (*n* = 40/group). **B** Schematic of the imaging protocol. Each cohort of mice was imaged at weeks 12, 15, 18, and 20 after starting their respective diets

**Fig. 2:**
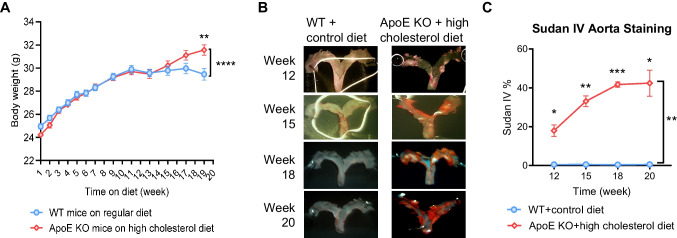
Establishment of atherosclerotic disease in an *Apoe*^−/−^ mouse model. **A** Longitudinal monitoring of the body weight of *Apoe*^−/−^ mice on a high-fat diet (HFD) compared with C57BL/6 control mice fed a regular chow diet for up to 20 weeks. **B** Representative Sudan IV red staining and (**C**) quantification of isolated aorta of *Apoe*^−/−^ and C57BL/6 mice after 12, 15, 18, and 20 weeks on respective diets confirms lipid accumulation in *Apoe*^−/−^/HFD group. Statistical significance was determined by two-way ANOVA, using Tukey’s multiple comparisons test. *p < 0.05; **p < 0.01; *** *p* < 0.001; ****p < 0.0001

To induce atherosclerotic disease 6–8-week-old *Apoe*^−/−^ mice (*n* = 40) were placed on a high fat diet (HFD) for up to 20 weeks and age-matched wildtype (WT) mice (*n* = 40) were placed on a regular chow diet. *Apoe*^−/−^ and WT mice underwent ^18^F-NaF or ^18^F-FDG PET imaging, followed by high-resolution microCT as illustrated in Fig. 1A at weeks 12, 15, 18, and 20. Following each imaging session (Fig. 1B), a subset (*n* = 3 per group) was euthanized, and the aorta was dissected postmortem. Disease progression was monitored by weekly body weight measurements (Fig. 2A) and disease presentation and severity were confirmed with postmortem Sudan IV staining (Fig. 2B). No significant difference in body weight was observed at early time-points. However, at 20 weeks on HFD, *Apoe*^−/−^ mice showed significantly higher body weight compared to WT control mice (*p* = 0.01) (Fig. 2A). Longitudinal ANOVA analysis combining the effect of group and time revealed a significant difference (*p* < 0.0001). Sudan IV staining of the intimal surface of the aorta revealed significant lipid accumulation in *Apoe*^−/−^ mice after 12 weeks on HFD (*p* = 0.004) compared to regular chow-fed WT mice (Fig. 2C). This trend of increased aortic Sudan IV staining continued into weeks 15 and 18 in *Apoe*^−/−^ mice (*p* < 0.0005) (Fig. 2C). Longitudinal ANOVA analysis revealed a significant difference (*p* = 0.0018).

**Fig. 3:**
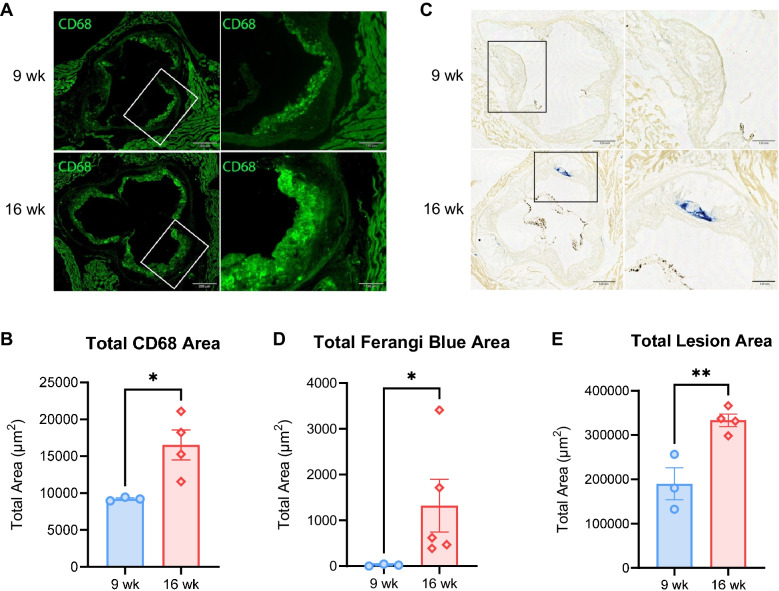
*Apoe*^*−/−*^/HFD mouse aortic root plaque histology analysis. **A** Representative fluorescent images of mouse aortic root plaque histology stained for CD68 in *Apoe*^*−/−*^/HFD mice at 9 and 16 weeks. Higher magnification images highlight CD68+ macrophage infiltration. **B** Quantification of total CD68+ area (µm^2^) at 9 and 16 weeks. **C** Representative images of aortic root sections stained with Ferangi Blue to assess calcification in *Apoe*^*−/−*^/HFD mice at 9 and 16 weeks. **D** Quantification of total Ferangi Blue-stained area (µm^2^) at 9 and 16 weeks. **E** Quantification of total lesion area (µm^2^) at 9 and 16 weeks of Ferangi Blue stained aortic root sections. Data are mean ± SEM, *N* = 3–5. Statistical significance was determined by unpaired two-tailed t-test. *, *p* < 0.05. **, *p* < 0.01

To investigate the abundance of the targets associated with calcification, which can be imaged with ^18^F-NaF PET and microCT, and macrophages in inflammatory atherosclerotic plaques assessed with ^18^F-FDG PET, we performed histology analyses of the aorta roots for CD68 expression and calcification using Ferangi Blue staining. As disease progressed, the total CD68-positive and calcified areas increased 79.7% (*p* = 0.030) (Fig. 3A and B) and 58.4-fold (*p* = 0.0357) (Fig. 3C and D) respectively. Additionally, the total lesion area (Fig. 3E), defined as the plaque area from the intimal edge to the border of the intima-media junction of all three cusps, increased 75.5% (*p* = 0.0088). These results further confirm disease progression in our *Apoe*^*−/−*^/HFD mouse model of atherosclerosis.

### Detection of atherosclerosis by in vivo ^18^F-NaF PET imaging

**Fig. 4:**
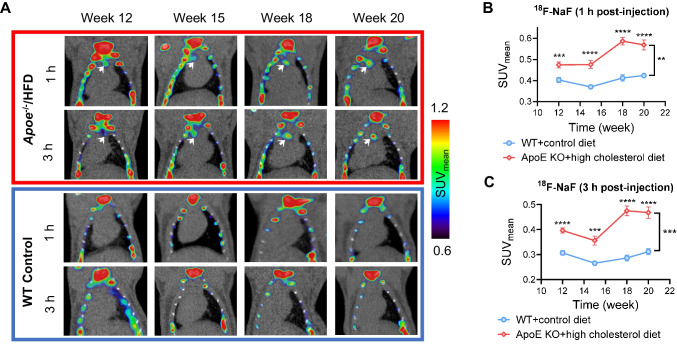
Detection of atherosclerosis by in vivo ^18^F-NaF microPET. **A** Representative coronal plane of the co-registered microPET/CT images of the *Apoe*^−/−^ and control mice at week 12, 15, 18, and 20 on their respective diets, 1 h and 3 h post injection of ^18^F-NaF. White arrows indicate ^18^F-NaF uptake in the aorta. **B** Quantitative PET of aortic ^18^F-NaF uptake in the *Apoe*^−/−^/HFD and WT control mice at week 12 (*n* = 20/group), 15 (*n* = 17/group), 18 (*n* = 14/group), and week 20 (*n* = 11/group), 1 h and (**C**) 3 h post injection of ^18^F-NaF. Statistical significance was determined by mixed-effect two-way ANOVA using Tukey’s multiple comparisons test. *p < 0.05; **p < 0.01; *** *p* < 0.001; ****p < 0.0001

After 12 weeks on their respective diets, *Apoe*^−/−^ and WT mice (*n* = 20/group) were imaged with ^18^F-NaF PET to detect the surface area of calcification. Mice were imaged 1 and 3 h post radiotracer injection (Fig. 4A). Quantitative analysis revealed a trend of increased ^18^F-NaF uptake in the aortas of *Apoe*^−/−^ mice compared to the control group after each imaging session (Fig. 4B-C). At the early 1 h timepoint, high amounts of circulating ^18^F-NaF contributed to the elevated background PET signal, with 11 of 20 mice presenting with hyperintense aortic ^18^F-NaF signals. This phenomenon was resolved when images were acquired at 3 h post tracer injection. As a result, 3 h post-injection was determined to be the optimal time point for ^18^F-NaF PET image acquisition. *Apoe*^−/−^ aortic calcification peaked at 18 weeks post initiation of HFD, evident by the substantive increase in aorta bound ^18^F-NaF compared to weeks 12 and 15. Significantly higher aortic ^18^F-NaF uptake was observed in the *Apoe*^−/−^ mice compared to the WT mice at all time points (*p* ≤ 0.0005; t-test). Longitudinal ANOVA analysis combining the effect of group and time also revealed a significant difference (*p* = 0.009).

### Detection of atherosclerosis in mice by ^18^F-FDG PET imaging

**Fig. 5:**
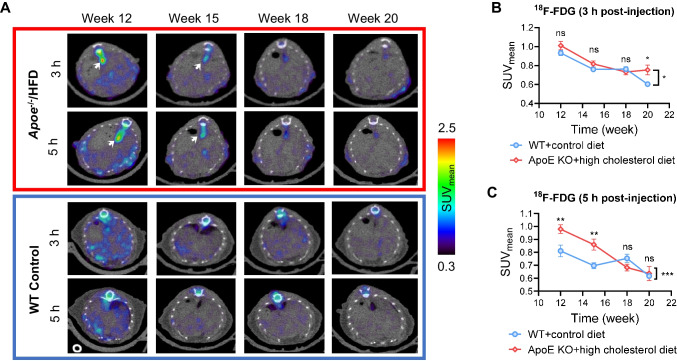
Detection of atherosclerosis by in vivo ^18^F-FDG microPET. **A** Representative transverse plane of the co-registered microPET/CT images of the *Apoe*^−/−^ and control mice at week 12, 15, 18, and 20 on their respective diets, 3 h and 5 h post injection of ^18^F-FDG. A white arrow indicates ^18^F-FDG uptake in the aorta. **B** Quantitative PET analysis obtained 3 h and (**C**) 5 h post-injection of ^18^F-FDG in *Apoe*^−/−^/HFD and WT control mice at week 12 (*n* = 20/group), 15 (*n* = 17/group), 18 (*n* = 14/group), and 20 (*n* = 11/group). Statistical significance was determined by mixed-effect two-way ANOVA, Tukey’s multiple comparisons test. *p < 0.05; **p < 0.01; *** *p* < 0.001; ****p < 0.0001; ns, not significant

A separate cohort of *Apoe*^−/−^ (*n* = 20) and WT (*n* = 20) mice were imaged with ^18^F-FDG PET/CT. Increased ^18^F-FDG uptake is a marker of atherosclerotic inflammatory disease through the enhanced glycolytic demand of activated immune cells [4]. Mice were imaged 3 h and 5 h post i.v. injection of ^18^F-FDG (Fig. 5A). While no significant difference was detected at 3 h post-injection (Fig. 5B), quantitative PET analysis revealed optimal aortic signal-to-noise was achieved at 5 h post-^18^F-FDG injection (Fig. 5C); significantly higher aortic uptake of ^18^F-FDG was observed in *Apoe*^−/−^ compared to WT mice. Peak aortic inflammation was observed at week 12 and decreased in weeks 15, 18, and 20 (Fig. 7). Beyond week 15, no statistically significant difference in aortic ^18^F-FDG PET signal was observed between *Apoe*^−/−^/HFD and WT mice (Fig. 5C). However, longitudinal ANOVA analysis combining the effect of group and time revealed a significant difference (*p* = 0.0006). Taken together, our data indicate that ^18^F-FDG PET imaging is only suitable for the diagnosis of atherosclerotic disease at the early stages.

### Detection of atherosclerosis in mice by microCT imaging

**Fig. 6:**
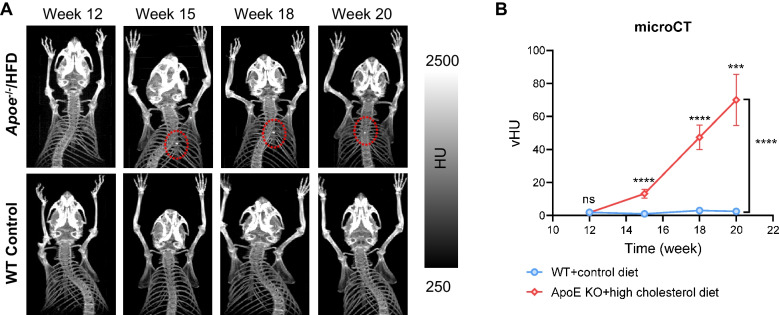
Detection of atherosclerosis by in vivo microCT. **A** Representative coronal view with maximum-intensity projection of the *Apoe*^−/−^ and control mice at week 12, 15, 18, and 20 on their respective diets. A dashed red circle indicates calcification observed by the microCT. **B** Quantitative analysis of aortic calcium content in the *Apoe*^−/−^/HFD and control mice at week 12 (*n* = 40/group), 15 (*n* = 34/group), 18 (*n* = 28/group), and 20 (*n* = 22/group). Statistical significance was determined by mixed-effect two-way ANOVA, using Tukey’s multiple comparisons test. *p < 0.05; **p < 0.01; *** *p* < 0.001; ****p < 0.0001; ns, not significant

Following ^18^F-NaF or ^18^F-FDG PET, mice received a high-resolution (100 μm) microCT scan to measure calcification (Fig. 6A). Based on previous studies, the CT HU value of > 300 HU can distinguish coronary artery plaques from surrounding myocardium (HU < 100) as such CT analysis and HU quantification was determined using ROI thresholding of > 350 HUs. Distinct from ^18^F-NaF, no statistical difference in dense calcification was observed at week 12 between *Apoe*^−/−^ and WT control mice (Fig. 6B), an observation that may be attributed to the presence of calcification mass (< 100 μm) that is below the CT limit of detection. However, by week 15 and up to week 20 (*n* = 22/group), significantly higher calcified mass was measured in *Apoe*^−/−^ mice. A significant increase in visible and measured calcification density was observed as the disease progressed (*p* < 0.001). Our results indicate that while CT lacks the sensitivity of PET to detect early-stage atherosclerotic disease, high-resolution CT can effectively monitor disease progression once the calcification reaches or exceeds the CT 100 μm detection limit.

## Discussion

Leveraging multiple imaging modalities, we longitudinally monitored metabolic glucose demand as a surrogate for inflammation with ^18^F-FDG PET, intimal calcification activity with ^18^F-NaF, and calcification density with CT in a preclinical model of atherosclerotic disease. Our goal in conducting this multi-cohort assessment was to identify the modalities that facilitate early diagnosis and enable monitoring of disease progression. While previous studies have reported these three imaging modalities as capable of detecting and/or monitoring disease progression, there has yet to be a systematic multi-cohort longitudinal assessment of how best to leverage each assay in the context of atherosclerosis progression stages [4].

In this study, we observed high accumulation of ^18^F-NaF and ^18^F-FDG in different regions, with ^18^F-NaF signal most pronounced in aortic arch and ^18^F-FDG accumulated in thoracic aorta. In WT mice, the absence of active calcification resulted in very low arortic ^18^F-NaF signal. ROIs were drawn by selecting the aortic arch region furthest from skeletal bones to minimize spillover. For ^18^F-FDG, uptake is high in the myocardium and the brown adipose tissue; the proximity to the aortic arch contributes to substantive signal spillovers that make accurate quantification challenging. Our findings are consistent with previous publications, where the aortic arch is the site of microcalcification or ^18^F-NaF PET accumulation while ^18^F-FDG PET is predominantly within the thoracic aorta of *Apoe*^*−/−*^/HFD mice [24, 25].

**Fig. 7:**
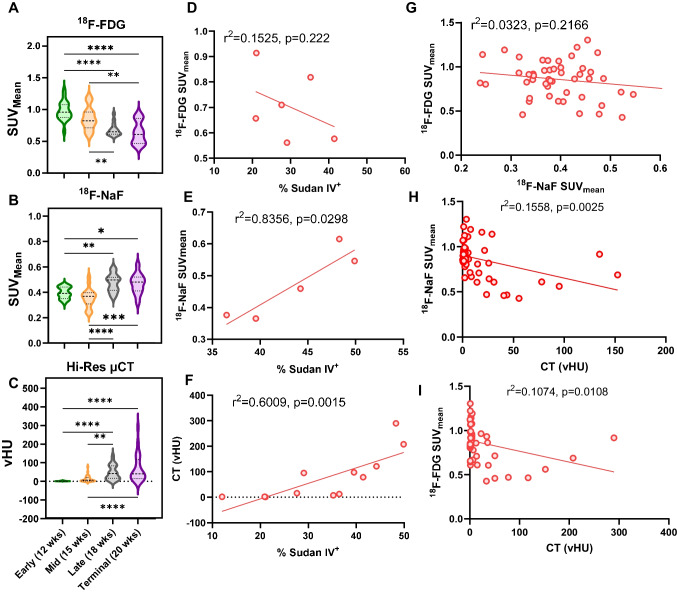
Multi-cohort molecular PET and anatomical CT imaging in an *Apoe*^−/−^/HFD model of progressive atherosclerosis. **A** Quantitative ^18^F-FDG, **B** ^18^F-NaF PET SUV, and **C** microCT in the *Apoe*^−/−^ mice fed a high-fat diet to induce atherosclerosis and monitored during early, mid, late, and terminal disease after 12, 15, 18, and 20 weeks of HFD, respectively. **D, E, F** Correlative analysis in matched subjects comparing ^18^F-FDG or ^18^F-NaF PET or anatomical high-resolution CT with terminal Sudan IV staining. A strong correlation between lipid accumulation (Sudan IV) and ^18^F-NaF PET or anatomical high-resolution CT was observed, while ^18^F-FDG did not correlate with %Sudan IV-positive area. **G, H, I** No correlation was observed with ^18^F-FDG or ^18^F-NaF SUV_mean_, while there was a strong correlation between microCT and ^18^F-FDG or ^18^F-NaF. Correlative analysis was conducted using the Pearson’s rank correlation coefficient. Statistical significance was determined by one-way ANOVA, Šídák's multiple comparisons test. *p < 0.05; **p < 0.01; *** *p* < 0.001; ****p < 0.0001

A defining characteristic of atherosclerosis is inflammation, and ^18^F-FDG PET imaging has been successfully utilized to detect inflammatory diseases and shows promise as a non-invasive tool for visualizing inflamed plaques in patients [5]. Similarly, in this preclinical study, we observed significantly elevated aortic ^18^F-FDG signal in the *Apoe*^−/−^ mice compared to WT mice. Pathologically, microscopic inflammation precedes plaque buildup in the arterial walls and is a hallmark of early disease and because of their elevated metabolic activities, macrophages consume glucose at a high rate [26]. Additionally, previous studies have found atherosclerotic ^18^F-FDG uptake strongly correlates with macrophage density infiltrated within the fibrous cap [4, 27, 28]. Interestingly, ^18^F-FDG PET signal peaked at week 12 (Fig. 7A) when the plaque burden (Fig. 2) was lowest (Fig. 7D). Additionally, we observed a 30% decrease in ^18^F-FDG PET signal intensity over time, and by week 18 there was no significant difference between diseased and control mice. Our findings highlight the transitory nature of atherosclerotic inflammation and further support reported findings where early-stage aortic accumulation of ^18^F-FDG is attributed to macrophage foam cell formation and at later stages of disease ^18^F-FDG PET signal decreased as a consequence of their differentiation [26, 29]. This transitory nature may explain why some patients with known risk factors may not exhibit abnormal tracer uptake, as atheromas may only show transient uptake patterns that are not amenable to imaging over extended periods in human subjects.

The use of ^18^F-FDG to visualize atherosclerotic plaques located on the aortic arch and thoracic aorta in live mice is challenging and further confounded by high uptake in the heart and brown adipose tissue in close proximity to diseased regions. Consistent with clinical reports, we identified the thoracic aorta as the primary site of inflammatory ^18^F-FDG PET signal [30]. The high spillover from the heart and adipose tissue may account for the modest or lack of differences observed between the *Apoe*^*-/-*^/HFD group and the WT control. Lastly, 5 h post-injection imaging was superior to 3 h post-injection, allowing for decreased ^18^F-FDG blood activity and improved our ability to detect early atherosclerosis. This observation is consistent with clinical observations where blood ^18^F-FDG activity has been reported to mask artery wall signals and hamper the detection of vascular inflammation [31]. Our study suggests delaying PET scan acquisition to ≥ 5 h after ^18^F-FDG injection may be warranted for more accurate delineation of vascular inflammation.

While ^18^F-FDG is taken up by metabolically active cells and serves as a surrogate for inflammation, ^18^F-NaF PET monitors a distinctly different hallmark of atherosclerosis. ^18^F-NaF identifies sites of active calcium deposition within vulnerable atherosclerotic lesions via the physicochemical exchange of hydroxyl groups of Ca-HAP with radioactive ^18^F^−^ ions [32, 33]. Our study revealed 3 h ^18^F-NaF uptake is required to achieve optimal signal-to-noise and reduce the rate of false positives due to incomplete clearance of the ^18^F-NaF tracer in circulation. Similar to atherosclerotic minipig studies where ^18^F-NaF signal was detected in regions of the vessel wall devoid of histological calcification [34], we attribute these false positives at the early 1 h post-injection time point to blood volume retention within the small vessel wall [35]. Images acquired at 1 h and 3 h post injection of ^18^F-NaF detected increased Ca-HAP presence at weeks 12 and 15 in *Apoe*^−/−^/HFD mice. Compared to control mice, *Apoe*^−/−^/HFD aortic ^18^F-NaF signal was 6- and ten-fold higher at weeks 12 and 15 respectively. This trend of increased ^18^F-NaF uptake persisted and peaked at week 18 (Fig. 7B). Lastly, our preclinical results are consistent with a recently published clinical ^18^F-FDG/^18^F-NaF dual-tracer PET/CT study in patients with carotid atheroma (NCT01724749); similarly, we observed no correlation between ^18^F-NaF and ^18^F-FDG PET (Fig. 7G). Additionally, we observed a strong correlation with increased ^18^F-NaF with disease progression/severity (*p* = 0.02, Fig. 7E), while clinically, ^18^F-NaF increased with elevated 10-year CVD risk (*p* < 0.01), but no significant trend was observed with ^18^F-FDG [36].

In addition to ^18^F-FDG and ^18^F-NaF, newer PET tracers are under investigation for better imaging atherosclerosis, such as CXCR4-targeted ^68^ Ga-pentixafor, CCR2-targeted ^64^Cu-DOTA-ECL1i, and CCR5-targeted ^64^Cu-DOTA-DAPTA [37]. Recently, an ^89^Zr-CD45 nanobody was reported as a robust PET tracer for imaging inflammation in multiple preclinical models [38]. While these tracers show promise, further robust comparative preclinical and clinical imaging studies are needed to inform how best these tools can be implemented for detecting and monitoring atherosclerosis.

Our preclinical evaluation of translational imaging methods for diagnosis and longitudinal monitoring of atherosclerosis shows that molecular PET imaging enables visualization of microscopic processes such as inflammation and Ca-HAP activity that precede morphological anatomical changes. Anatomical CT is efficient for the visualization of dense calcified deposits. In our preclinical studies, calcified lesions were not detectable by microCT until mid-late stages of the disease (Fig. 7C). In fact, no statistical differences in CT HU values were observed between *Apoe*^−/−^ and control mice at week 12. However, from week 15 onwards, the CT HU values steadily increased over time. Similar to ^18^F-NaF, we observed a strong correlation between CT HU and disease severity (Fig. 7F). Furthermore a strong correlation was observed between CT HU and both ^18^F-FDG and ^18^F-NaF PET (Fig. 7H,I).

## Conclusion

The results of this multi-cohort preclinical study reveal ^18^F-FDG PET and ^18^F-NaF PET are more effective at detecting early atherosclerotic lesions. Of the two PET assays, only ^18^F-NaF PET could monitor progressive disease. Importantly, ^18^F-NaF PET was capable of detecting disease earlier (week 12) than was achieved with anatomical microCT. However, as the disease progressed into later stages (weeks 18–20) microCT proved more effective than ^18^F-NaF PET. In summary, we have demonstrated the pairing of molecular and anatomical imaging provides a comprehensive overview of atherosclerotic pathobiology, whereby ^18^F-FDG PET is best used to measure inflammatory disease prevalent in early disease while ^18^F-NaF PET serves as an early readout of Ca-HAP activity that results in dense calcified lesions which are best visualized by CT, especially during late-stage disease. Importantly, we observed a strong correlation between ^18^F-NaF (r^2^ = 0.83, *p* = 0.0298) and anatomical CT (r^2^ = 0.60, *p* = 0.0015) with disease severity. Given the prevalence of integrated PET/CT instruments for the comprehensive detection of disease or monitoring of response to therapeutic interventions, molecular imaging of atherosclerotic inflammation (^18^F-FDG) and Ca-HAP activity (^18^F-NaF) should be coupled with high-resolution anatomical CT.

## Data Availability

The datasets generated during the study are available from the corresponding author on reasonable request.

## Abbreviations

microCT: Micro-computed tomography
microPET: Micro-positron emission tomography
^18^F-NaF: Sodium fluoride radiolabeled with fluorine-18
^18^F-FDG: Fluorodeoxyglucose radiolabeled with fluorine-18
*Apoe*^−^^/^^−^: Apolipoprotein E deficient
CVD: Cardiovascular disease
Ca-HAP: Calcium hydroxyapatite
HFD: High fat diet
LDL: Low-density lipoprotein
